# *Aspergillus Carneus* metabolite Averufanin induced cell cycle arrest and apoptotic cell death on cancer cell lines via inducing DNA damage

**DOI:** 10.1038/s41598-023-30775-w

**Published:** 2023-04-20

**Authors:** Deren Demirel, Ferhat Can Ozkaya, Weaam Ebrahim, Emel Sokullu, Irem Durmaz Sahin

**Affiliations:** 1 Koc University Research Center for Translational Medicine (KUTTAM), Sariyer, Istanbul, Turkey; 2 Aliaga Industrial Zone Technology Transfer Office, Aliaga, 35800 İzmir, Turkey; 3 Department of Pharmacognosy, Faculty of Pharmacy, Mansoura University, Mansoura, 35516 Egypt; 4 Koc University, School of Medicine, Sariyer, 34450 Istanbul, Turkey

**Keywords:** Cancer, Drug discovery, Molecular biology

## Abstract

Cancer is one of the leading causes of death worldwide, accounting for nearly 10 million deaths in 2020. Current treatment methods include hormone therapy, γ-radiation, immunotherapy, and chemotherapy. Although chemotherapy is the most effective treatment, there are major obstacles posed by resistance mechanisms of cancer cells and side-effects of the drugs, thus the search for novel anti-cancer compounds, especially from natural sources, is crucial for cancer pharmaceutics research. One natural source worthy of investigation is fungal species. In this study, the cytotoxicity of 5 metabolic compounds isolated from filamentous fungus *Aspergillus Carneus.* Arugosin C, Averufin, Averufanin, Nidurifin and Versicolorin C were analyzed using NCI-SRB assay on 10 different cell lines of breast cancer, ovarian cancer, glioblastoma and non-tumorigenic cell lines. Averufanin showed highest cytotoxicity with lowest IC_50_ concentrations especially on breast cancer cells. Therefore, Averufanin was further investigated to enlighten cell death and molecular mechanisms of action involved. Cell cycle analysis showed increase in SubG1 phase suggesting apoptosis induction which was further confirmed by Annexin V and Caspase 3/7 Assays. H2A.X staining revealed accumulation of DNA damage in cells treated with Averufanin and finally western blot analysis validated DNA damage response and downstream effects of Averufanin treatment in various signaling pathways. Consequently, this study shows that Averufanin compound induces cell cycle arrest and cell death via apoptosis through causing DNA damage and can be contemplated and further explored as a new therapeutic strategy in breast cancer.

## Introduction

Cancer is among the leading causes of death worldwide, accounting for around 10 million deaths in 2020^1^ In 2018, there were 18.1 million new cases and 9.5 million cancer-related deaths worldwide. By 2040, the number of new cancer cases per year is expected to rise to 29.5 million and the number of cancer-related deaths to 16.4 million^2^ To be able to restrain this probable outcome, investigation of new treatment strategies is vital.

Cancer treatment today includes, surgery, radiation therapy, hormone therapy, immunotherapy, targeted therapy, photodynamic therapy, and the most widely used one, chemotherapy^3^. Although chemotherapy is one of the most effective treatment options, acquired resistance of cancer cells remains as an obstacle to overcome^4^. Therefore, discovery of new anti-cancer compounds to be used as chemotherapeutic agents is still an attractive field^5^.

Resistance mechanisms may vary between patients and some of the identified mechanisms can be listed as, inactivation of drugs, alterations of drug metabolism, inhibition of cell death, epigenetic altering, amplification or changing of target genes, apoptosis pathway blocking and enhancing DNA damage repair^6^. Cancer cells can also get multidrug resistance (MDR), through increasing efflux mechanisms and decreasing intake rate^7^. One of the important elements in cancer emergence, progression and resistance mechanisms are tumor suppressor genes. A well-known and widely studied example is TP53 gene which is found mutated in 50% of human cancers^8^. Protein p53 is most frequently activated in response to DNA damage; and cell-cycle arrest followed by apoptosis are the most prominent outcomes of its activation^9^. It functions through activating cyclin dependent kinase inhibitors such as p21^Cip1^ and apoptotic proteins such as Bax, PUMA and Noxa, respectively^10^. Although these are the prevalent roles of p53, studies show that these are not the only pathways p53 is involved. Some examples are p53 and lncRNA interactions^11^, miRNA processing^12^ and CyclinA2/CDK2 complex activating. The latter was recently demonstrated by a study that Cyclin A2/CDK2 complex is not just an S phase cell cycle regulator but also a DNA damage response and takes a role in double-strand repair coupling with Ku proteins^13^. The mechanisms activating p53 is also widely studied and among several other proteins in regulatory roles, GSK3β phosphorylates p53 so that it is in its active form^14^. GSK3β is also activated under stress and DNA damage conditions, upon dephosphorylation and is also capable of downregulating Cyclin D1 along with the downstream p53-p21-Cyclin D1 pathway^15^. In the presence of an active GSK3β, Cyclin D1 levels decrease affected by both pathways. Even though treatment options emerged rapidly and promisingly in the last years in breast cancer, patients acquire resistance quickly in most of the cases. Due to the status of its receptors, the susceptibility to alternative therapy methods, such as hormonal therapy, can be altered. For instance, an Estrogen Receptor (ER) positive cell population can be treated with drugs like Tamoxifen, only until the cells gain resistance and bypass receptor related therapies. In addition, there are different types of breast cancer classifications due to their receptor expressions such as Triple Negative Breast Cancer (TNBC) cells, HER2/ER/PR negative or positive cells. On the other hand, breast cancer possesses p53 mutations lower than other types of cancer according to several statistical data^10^.

This study investigated the cytotoxic effects of 5 compounds isolated from filamentous fungus *Aspergillus Carneus. Aspergillus Carneus* is primarily a soil fungus that colonizes mostly in tropical or subtropical terrestrial environments but also found and can grow worldwide^16^. Recent studies also found the species in marine environments^17–19^. Anti-cancer effects of fungi metabolites is a broad area of research and different molecules have been tested in different cancer cell lines including lung fibroblast cells^19^, pancreatic cancer cells^20^, liver cancer cells^21^ and more. Marine fungi secondary metabolites such as the ones in the current study have been previously reported as potential anti-cancer^22^, anti-microbial or anti-oxidant^23^ compounds however, there are a large variety of compounds from different species remains to be identified and their effects to be investigated. *Aspergillus spp.* renders many metabolites, some of which are tested for their cytotoxicity on some tumorigenic cell lines and their respective IC_50_ values are reported and reviewed in many studies^24–27^. There is a study showing anti-tumor growth effects of compound Aspergiolide A isolated from filamentous fungus *Aspergillus Glaucus* in mice xenograft model^27^. Bioactive metabolites from the species *Aspergillus Carneus* has not been extensively studied and reported for their anticancer activity and more importantly, other than its isolation and classification there is no publication to date on the anti-cancer, anti-microbial or anti-oxidant activity of Averufanin which makes this study novel in the area.

In this study, 5 chemical compounds were isolated from *Aspergillus Carneus* and tested against human cancer cell lines. We documented that Averufanin was highly cytotoxic towards breast cancer cells and relatively low toxicity on non-tumorigenic cell line. Treatment with Averufanin resulted in DNA damage, cell cycle arrest and apoptotic cell death on breast cancer cell lines by modulating p53 mediated cell signaling pathways. Therefore, Averufanin can be a novel candidate compound for therapeutic strategies in cancer research, especially holding a potential for breast cancer.

## Results

### Chemistry

**Figure 1:**
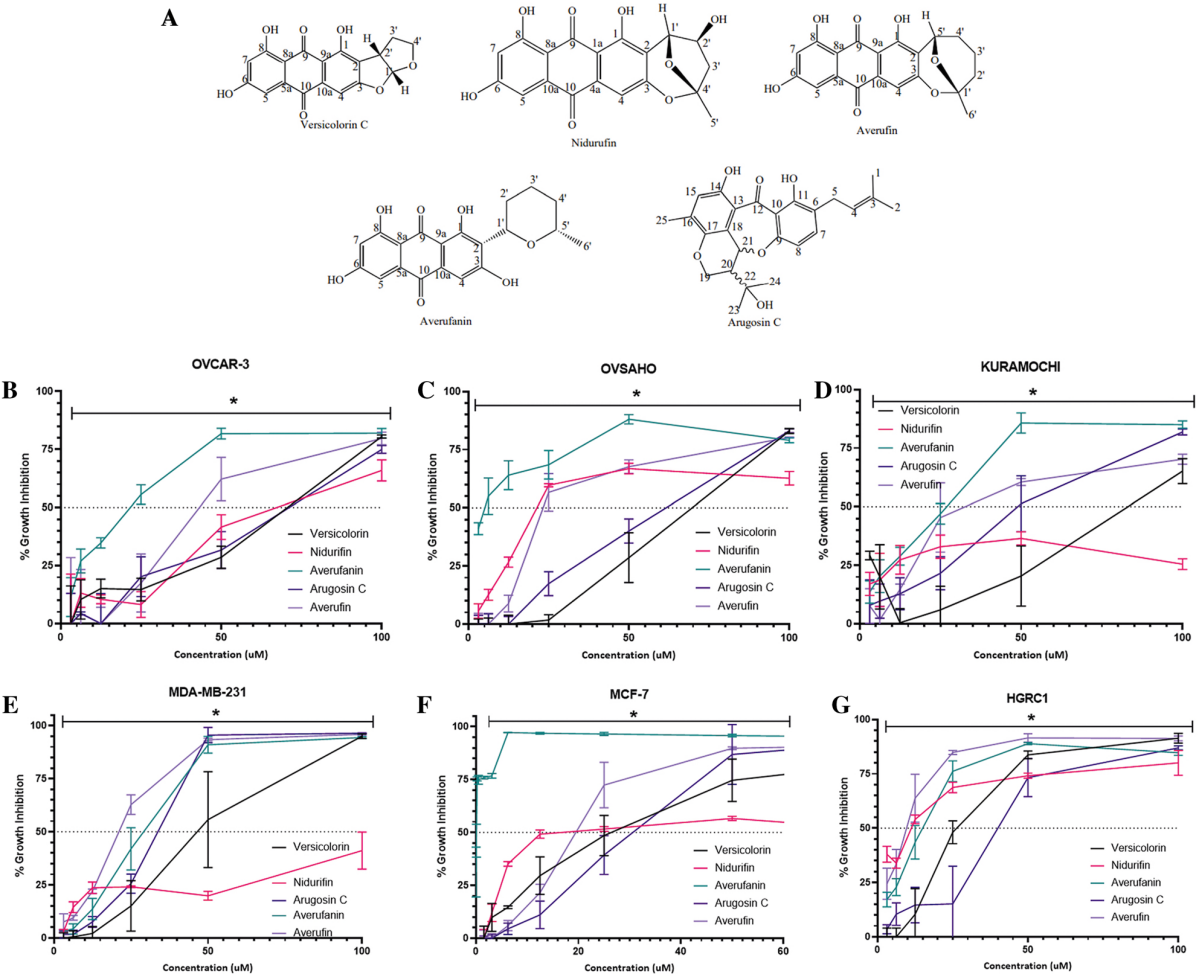
Bioactivities of the compounds isolated from *Aspergillus Carneus.* (**A**) Chemical structures of the isolated molecules drawn in ChemDraw. Percent growth inhibition curves were characterized by NCI SRB assay on ovarian ((**B**) OVCAR3, (**C**) OVSAHO, (**D**) KURAMOCHI) and breast ((**E**) MDA-MB-231, (**F**) MCF-7) cancer cell lines or non-tumorigenic cell line ((**G**) HGRC1) treated with the compounds for 72 h in increasing concentrations (0.2–100 µM). The experiment was conducted in triplicate and absorbance values were normalized to DMSO negative controls. Significantly difference compared to DMSO treated cells (*p < 0.05).

All chemicals were isolated from *Aspergillus Carneus* species, and their chemical formulas were drawn using ChemDraw is shown in Fig. 1A.

### Biology

#### Cytotoxicity evaluation of compounds on cancer cell lines

Cytotoxic activities of purified compounds obtained from *Aspergillus Carneus* (Fig. 1A) were initially investigated on breast cancer (MCF7, MDA-MB-213), ovarian cancer (OVCAR3, OVSAHO, KURAMOCHI) cell lines and a non-tumorigenic cell line from gynecological (HGRC1) origin using NCI SRB assay.

**Figure 2:**
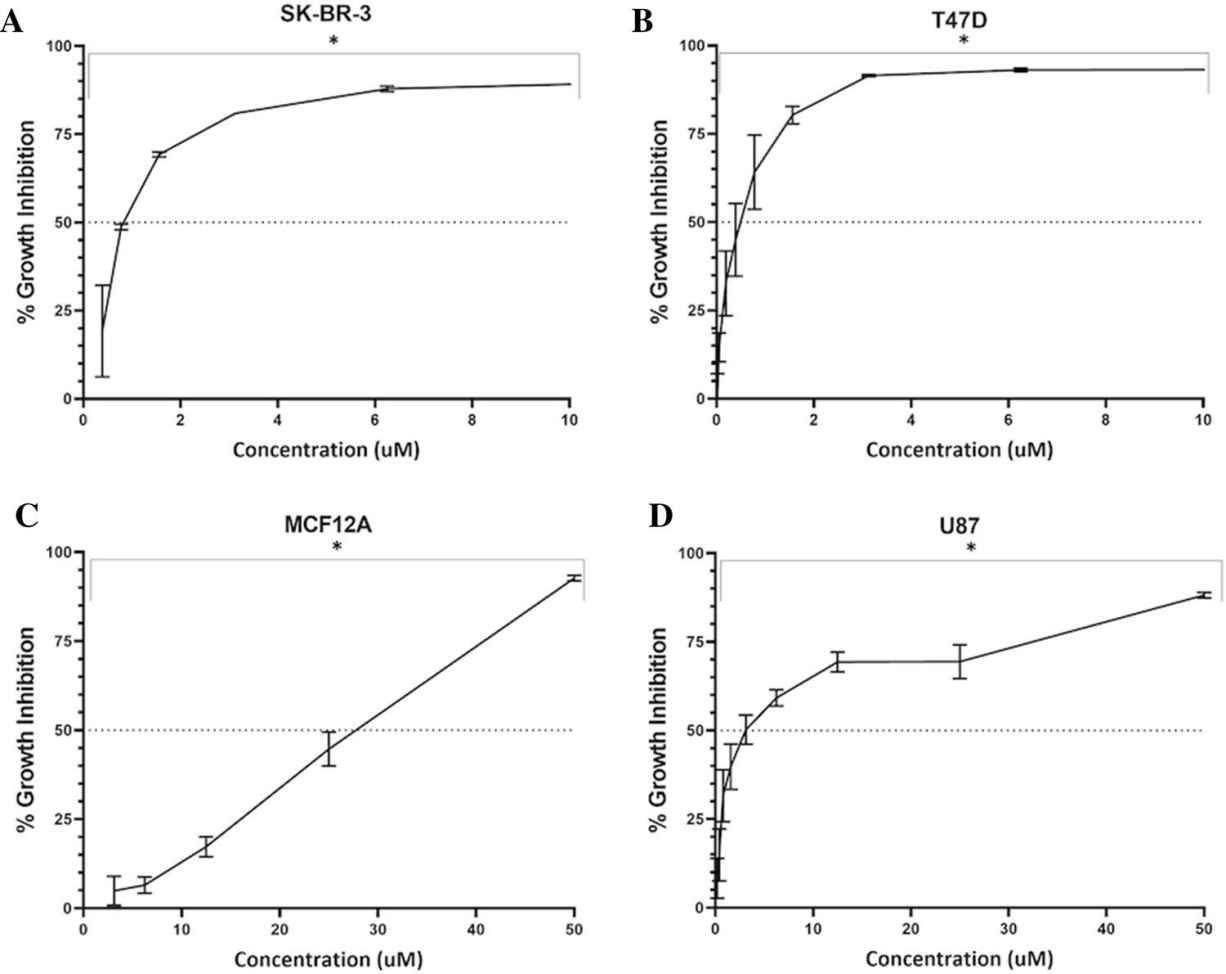
Anti-growth activity of Averufanin on additional cancer cell lines. Percent growth inhibition curves were characterized by NCI SRB assay on breast cancer (0.2–100 µM). The experiment was conducted in triplicate and absorbance values were normalized to DMSO negative controls. Significantly difference compared to DMSO treated cells (*p < 0.05).

From all 5 compounds isolated from *Aspergillus Carneus*, Averufanin showed significant levels of cytotoxicity whereas other molecules had no notable effects on cell growth (Fig. 1B–G and Table 1). These results indicated Averufanin as the promising compound for targeting cancer cell lines more effectively than the rest of the compounds and breast cancer cells were more sensitive to treatment with Averufanin therefore in the next panel of NCI-SRB assays, two additional breast cancer (T47D, SK-BR-3) and a Glioblastoma cell line U-87 were included as well as non-tumorigenic breast epithelial (MCF12A) cell line. Figure 2 shows that all three cancer cell lines (U-87, T47D, SK-BR-3) were significantly more sensitive to Averufanin treatment than non-tumorigenic MCF12A cell line.

**Table 1:** IC_50_ values of the compounds obtained from NCI-SRB Assay. *R*^2^ > 0.8.

	Cell line	Averufanin IC_50_ (µM)	Nidurifin IC_50_ (µM)	Versicolorin C IC_50_ (µM)	Averufin IC_50_ (µM)	Arugosin C IC_50_ (µM)
Ovarian	OVSAHO	5.27 ± 1.4	30.8 ± 1.4	> 40	31.9 ± 9.0	> 40
	OVCAR-3	17.2 ± 2.2	> 40	> 40	> 40	> 40
	Kuramochi	19.4 ± 2.4	> 40	> 40	38.6 ± 4.5	> 40
	HGRC1	12.6 ± 1.6	9.99 ± 1.1	29.5 ± 0.8	6.63 ± 1.6	33.5 ± 8.6
Breast	MCF-7	0.28 ± 0.1	39.1 ± 10.6	22.3 ± 10.8	20.8 ± 1.8	25.4 ± 11.9
	MDA-MB-231	30.6 ± 3.0	> 40	39.7 ± 13.4	18.5 ± 1.4	25.7 ± 3.5
	T47D	0.46 ± 0.1	–	–	–	–
	SK-BR-3	1.74 ± 0.5	–	–	–	–
	MCF12A	21.5 ± 1.7	–	–	–	–
Glioblastoma	U-87	3.63 ± 1.5	–	–	–	–

#### Cell cycle analysis with PI staining and flow cytometry

**Figure 3:**
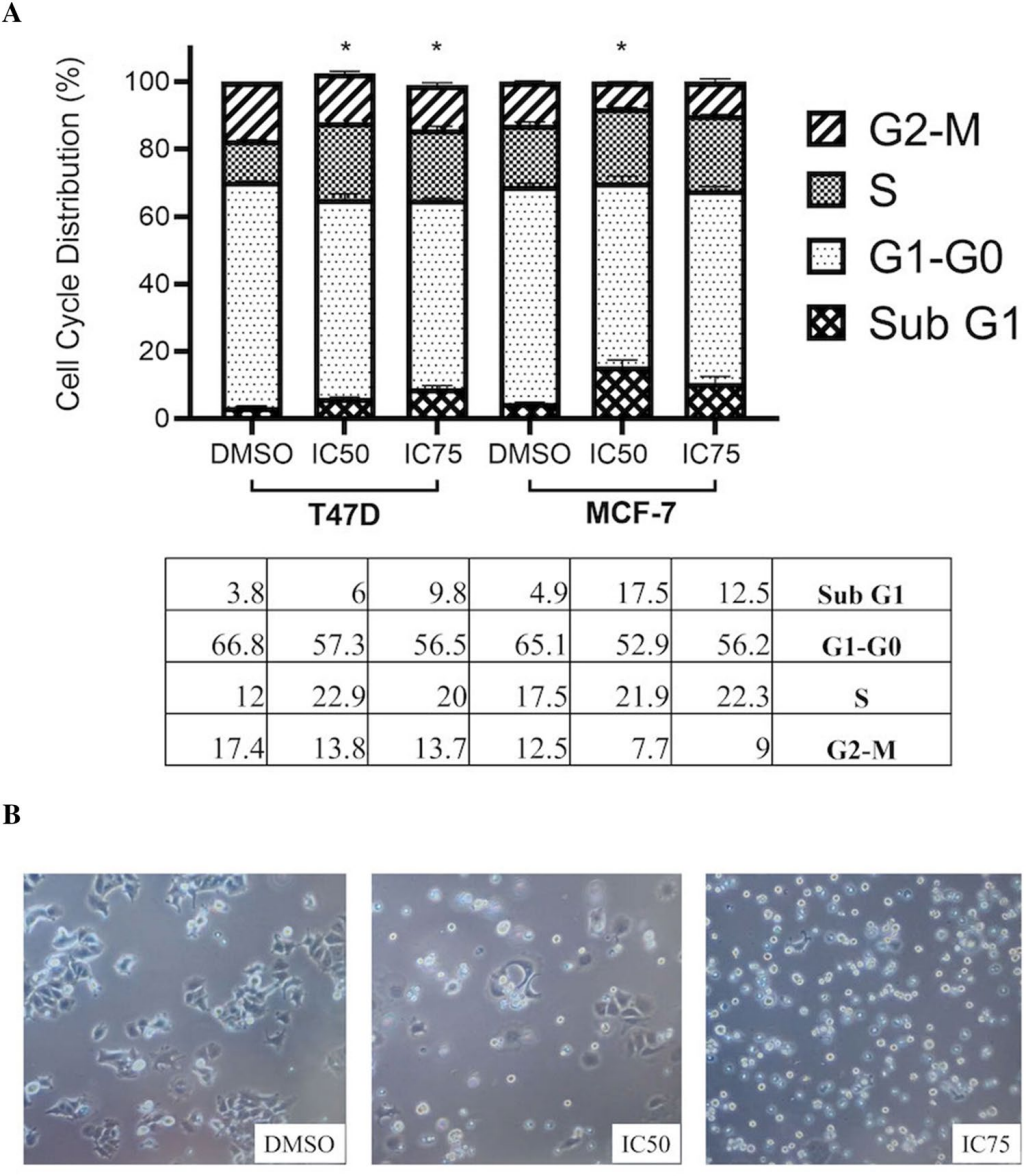
Cell cycle arrest and cell morphology changes induced by Averufanin. Human breast cancer cell lines MCF7 and T47D were treated with Averufanin or DMSO controls for 48 h and analyzed with flow cytometry. Bar graph shows the cell cycle stage comparison of control and treatment groups. Both treatment groups showed a significant SubG1 increase. Difference was significant compared to DMSO treated cells for SubG1 (**A**) (*p < 0.05) The brightfield microscopy image shows MCF-7 cell line treated with its respective DMSO Control, IC_50_ and IC_75_ doses. (×10 magnification) (**B**) 1 × 10^5^ cells were seeded in 6-wells for PI Cell Cycle Assay, images were taken before collection of cells. Cells undergoing apoptosis looks detached from the surface and spherical, whereas attached cells showed no apparent morphology change.

To further elucidate the effect of Averufanin on cell cycle, breast cancer cells were treated with Averufanin (IC_50_ or IC_75_) or DMSO negative controls for 48 h and stained with a DNA dye, propidium iodide (PI). Propidium iodide, which is a DNA-intercalating fluorescent dye, is frequently used for cell cycle analysis. Cells that are in G1 phase of cell cycle or at quiescent state (G_0_) contain one DNA copy (2N), whereas cells at G2/M phase have double copies of DNA (4N). Moreover, if the cells are in S phase of the cell cycle, they have varying copy number. Healthy cancer cells would have about 60–70% of the cells to be in G1 phase, 20% in S phase. Moreover, G2/m phase cells would comprise 20% of the total cell population. During apoptosis, one of the cellular responses is DNA fragmentation into smaller pieces, which is also visible in a PI Flow Cytometry assay as the “SubG0/G1” phase. Experiment results from this assay showed significant increase in SubG1 phase in cells treated with Averufanin compared to DMSO negative controls on both cell lines (Fig. 3A) which also suggested apoptotic cell death induction in those cells which was further analyzed with Annexin V and caspase activities in the following experiments. To further show cell death and cell morphology changes induced, brightfield microscopy images of cells under treatment were included. (Fig. 3B) The images are representative of MCF-7 cell line treated with 48 h DMSO negative control, IC_50_ and IC_75_ for cell cycle analysis. (10X magnification) Due to the effect of Averufanin it was captured within 48 h that respective population of cells are detaching from the surface looking spherical. The live, attached portion of cells show no significant morphology changes.

#### Characterization of cell death induced by Averufanin

**Figure 4:**
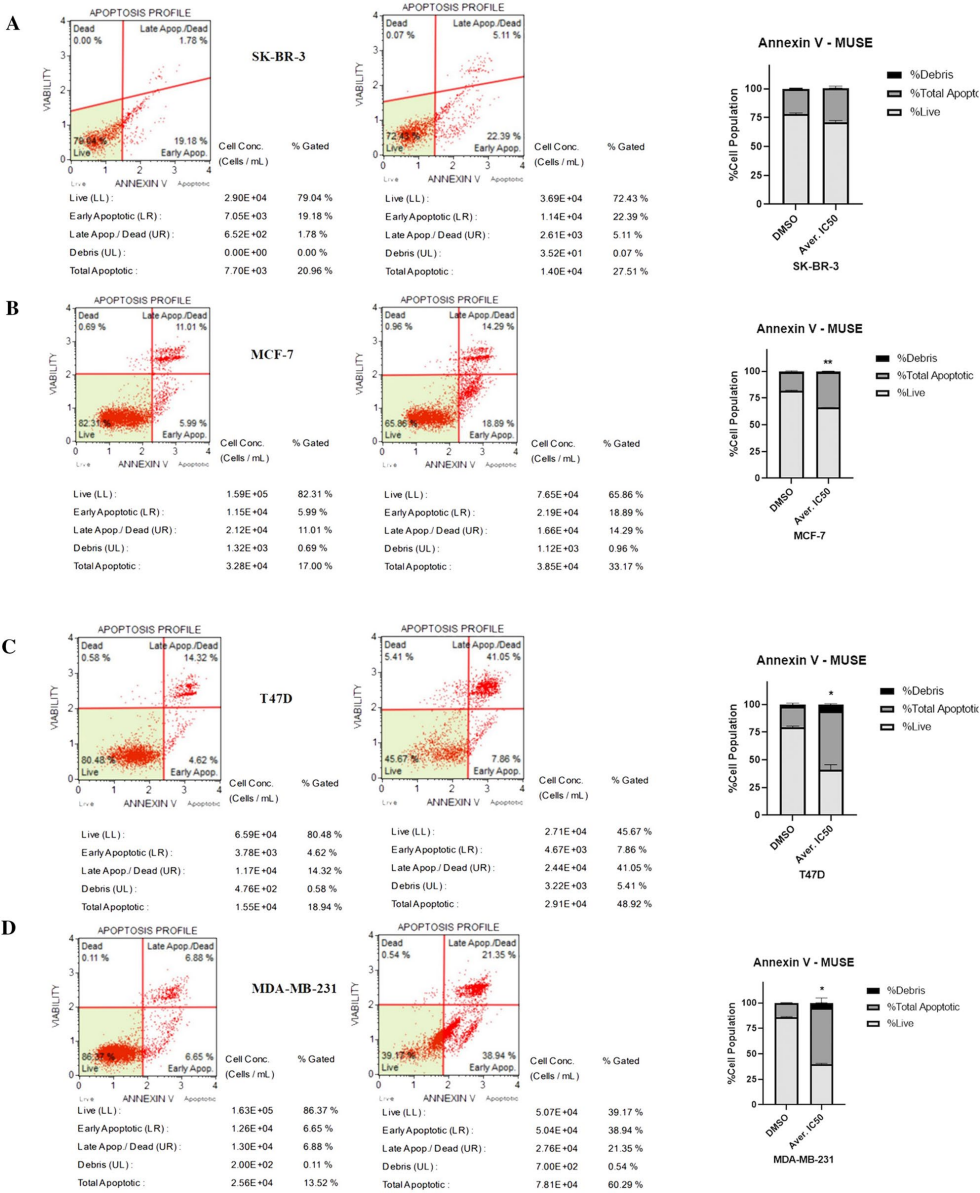
Averufanin induced accumulation of early and late apoptotic cells. The effects of Averufanin (IC_75_) treatment (right lane) on cancer cell lines (**A**) SK-BR-3, (**B**) MCF-7, (**C**) T47D, (**D**) MDA-MB-231 compared to DMSO negative control (left lane) for 72 h were investigated with MUSE Cell Analyzer using Annexin V & Dead Cell program and kit. Representative flow cytometry plots are shown. 10.000 cells were scored in each analysis. % cells in each quadrant are indicated on the plots. Treatment of each cell line resulted in a significant increase in the total apoptotic number of cells, majorly late apoptotic in T47D and majorly early apoptotic in MCF7. The results of Annexin V Assay suggested that Averufanin induced apoptosis in all three breast cancer cell lines. Significantly difference compared to DMSO treated cells (***p < 0.001, p < 0.01, p < 0.05).

To determine the cell death mechanism induced, MUSE Cell Analyzer was used. Human breast cancer cells were treated with Averufanin at their respective IC_75_ values for 48 h and DMSO was used as negative control. Annexin V & Dead Cell analysis shows cells positive for Annexin V, a marker for apoptosis on the outer membrane of the cells. Figure 4 shows early and late apoptotic breast cancer cells when treated with Averufanin.

**Figure 5:**
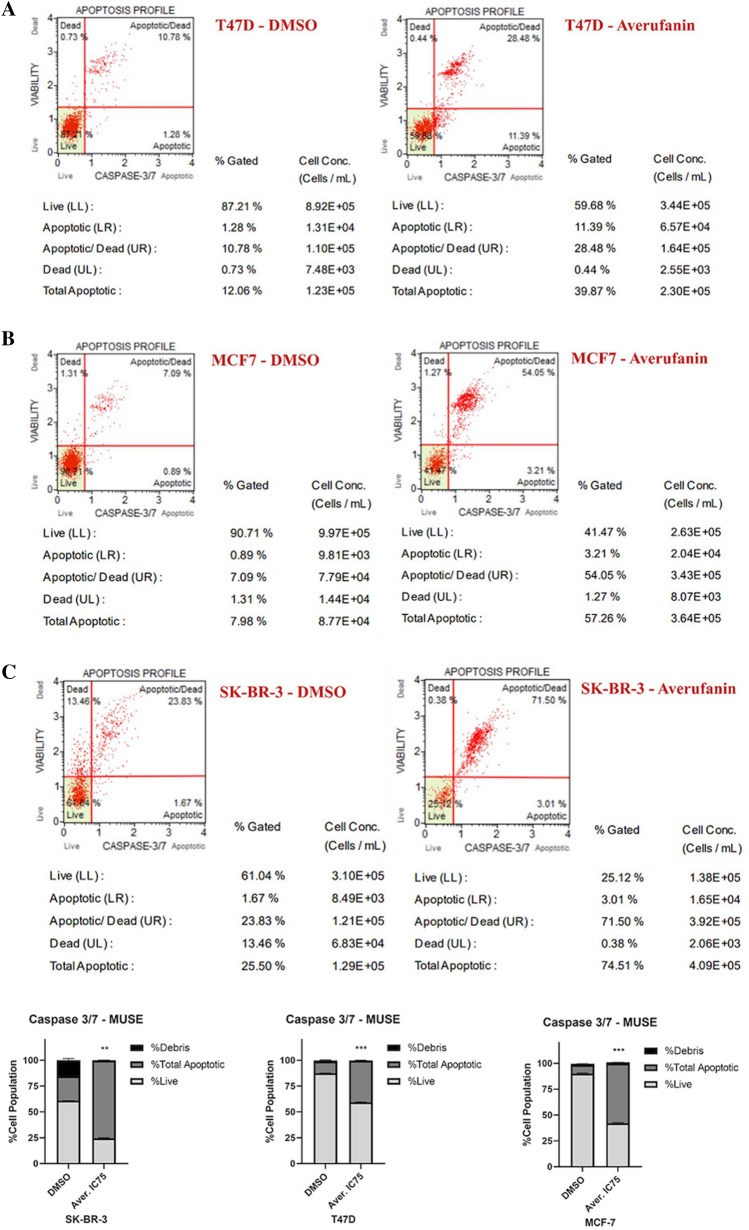
Increase in Caspase 3/7 activity upon treatment with Averufanin. The activities of caspases 3 and 7 of cancer cell lines (**A**) T47D, (**B**) MCF-7, (**C**) SK-BR-3 were analyzed upon treatment with Averufanin (IC_75_) (right lane), compared to DMSO negative control (left lane) for 48 h using MUSE Cell Analyzer using Caspase 3/7 program and kit. Averufanin treatment of all the cell lines showed a remarkable increase in the total percentage of apoptotic cells. Caspase 3/7 Assay indicated that Averufanin induced apoptosis, via activating caspase3/7 cascade. Significantly difference compared to DMSO treated cells (***p < 0.001 **p < 0.01, *p < 0.05).

In order to further assess the effect of Averufanin on apoptotic cell death, the activities of apoptosis pathway proteins Caspases 3 and 7 were investigated using MUSE Cell Analyzer Caspase3/7 Assay. Breast cancer cells were treated with Averufanin (IC_75_ concentrations, Table 1) or DMSO negative control for 48 h. Cells were later stained for caspase 3 and 7 and analyzed. Figure 5 showed that treatment of (A) T47D, (B) MCF-7, and (C) SK-BR-3 cells with Averufanin resulted in occurrence of more apoptotic cells than with DMSO controls.

#### Accumulation of DNA damage

**Figure 6:**
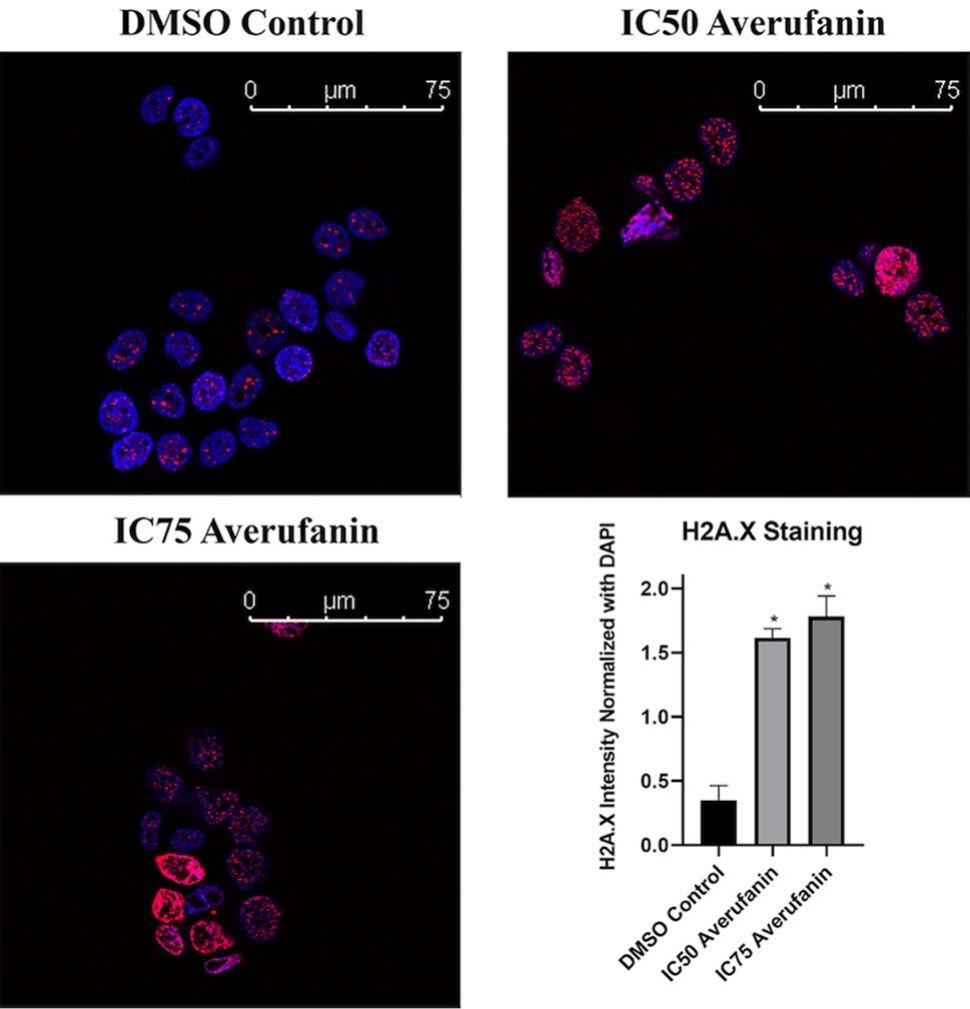
γ-H2Ax staining showed accumulation of DNA damage upon Averufanin treatment. Human breast cancer cells (T47D) were treated with (**A**) DMSO, (**B**) IC_50_ dose Averufanin, or (**C**) IC_75_ dose Averufanin for 48 h and stained with anti-γ-H2Ax and DAPI. Blue represents DAPI and red represents γ-H2Ax which increases as a DNA damage response (DDR). Images were obtained from the confocal microscopy through LasX and dye intensities were evaluated with ImageJ. H2A.X intensities were normalized to DAPI, graphics and statistical analysis were conducted with GraphPad PRISM. Two-tailed, unpaired t-test was applied between DMSO Control and treatment groups (*p < 0.05).

Phosphorylation of histone H2AX (γ-H2Ax) is a well-established marker of accumulation of dsDNA breaks. Therefore, human breast cancer cells were treated with Averufanin or DMSO negative control for 48 h and then stained with both DAPI and γ-H2Ax antibody, blue and red respectively. Our results indicated that Averufanin treatment resulted in accumulation of γ-H2Ax positive cells when compared to DMSO negative controls (Fig. 6).

#### Cellular pathways targeted by Averufanin

**Figure 7:**
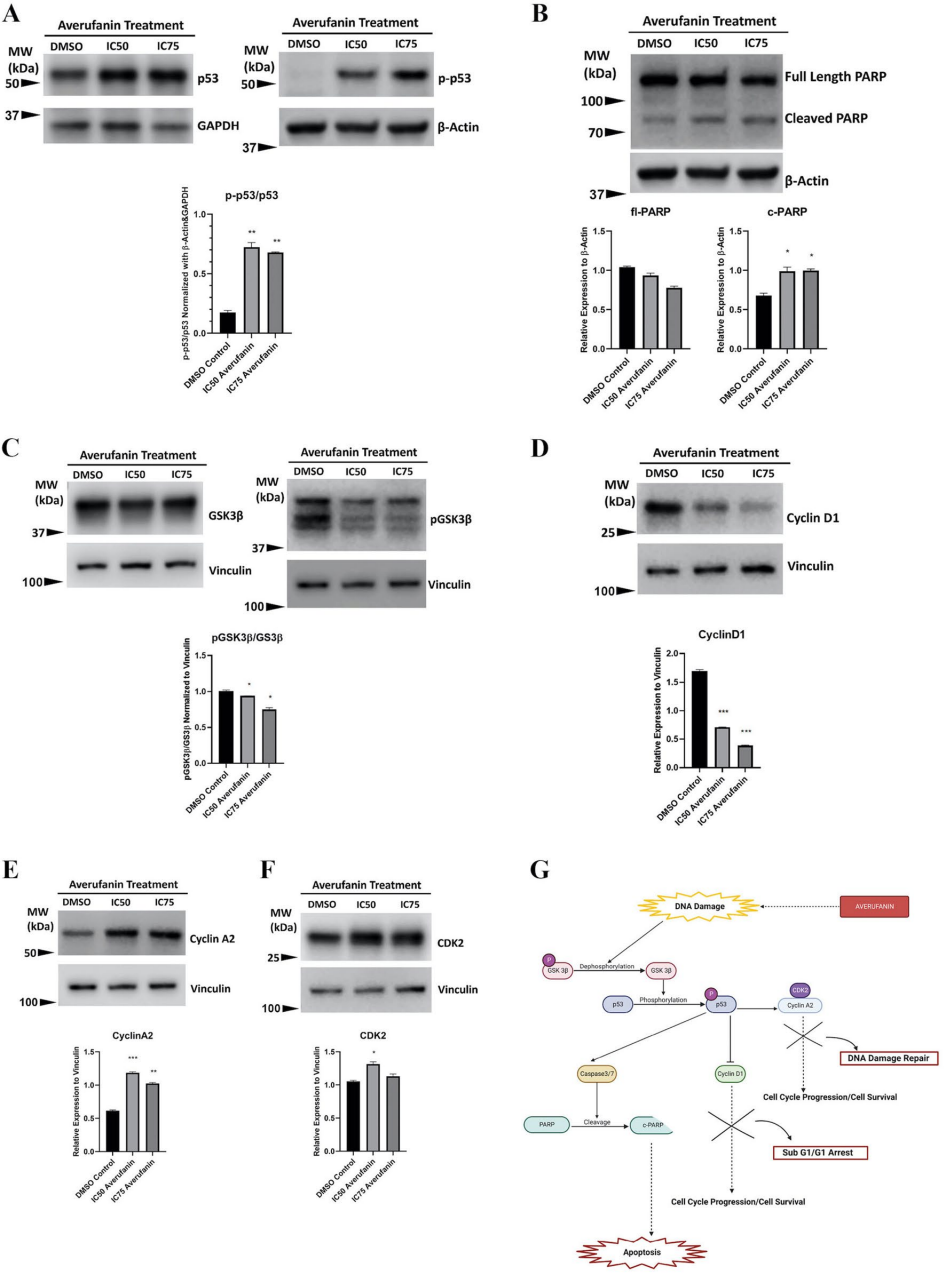
Cellular pathway components targeted by Averufanin treatment. Human breast cancer cells (T47D) were treated with the IC_50_ and IC_75_ concentrations of Averufanin or DMSO control for 48 h. (**A**) Western blot analysis showed that p53 protein was activated by phosphorylation upon treatment with Averufanin. Furthermore, (**B**) PARP protein was shown to be cleaved indicating induction of apoptosis. (**C**) pGSK3β and (**D**) Cyclin D levels were decreased in correlation with the cell cycle analyses. (**E**) Cyclin A2 and (**F**) CDK2 levels were shown to be increased in correlation with DNA damage response. Original blots are presented in Supplementary Figures. Quantification of the results were obtained using ImageJ software. Graphics and statistical analysis were performed with GraphPad PRISM, DMSO vs Treatment groups and p values are in the brackets. (**G**) Schematic representation of the molecular mechanism of action signaling of the compounds. Blocked pathways are crossed. Multi-step downstream effects are represented with dashed arrows. γ-H2Ax staining results indicated DNA damage caused by the compound, followed by dephosphorylation of pGSK3β and phosphorylation of p53. DNA damage induced activation of tumor suppressor p53 activates Caspase 3/7 pathway followed by PARP cleavage, along with CDK2-Cyclin A2 cell cycle proteins for DNA repair. At the same time Cyclin D1 is inhibited to stop the cell cycle until DNA damage can be repaired. (***p < 0.001 **p < 0.01, *p < 0.05 ns p < 0.5).

Based on the previous data on apoptotic induction, initially proteins involved in apoptosis pathway such as PARP, or p53 were analyzed. Western blot analysis on breast cancer cells treated with Averufanin reveled increase in phosphorylation of p53 (Fig. 7A) and in cleavage of PARP (Fig. 7B) compared to DMSO controls which supports induction of apoptosis. Furthermore, GSK3β and p-GSK3β was investigated to see if Averufanin treatment affected its phosphorylation. Dephosphorylation of Ser9 residue, which makes GSK3β active was observed. (Fig. 7C). Based on the flow cytometry results indicating cell cycle arrest, effect of the compound on Cyclin D1, which is a cell cycle regulator protein and an active GSK3β target was also investigated, and results showed significant decrease in Cyclin D1 levels upon treatment with Averufanin compared to DMSO controls (Fig. 7D). Another DNA damage response and cell cycle regulatory protein Cyclin A2 and its cyclin dependent kinase CDK2 was analyzed, and their levels showed an apparent increase, consistent with the DNA damage response expected. (Fig. 7E,F).

## Discussion

In 2020 Breast cancer was the world’s most prevalent cancer and deaths caused by it endures to be at an important rate even with all the treatment options. Therefore, search for novel anti-cancer compounds keeps its importance. One of the natural sources worthy of investigating is fungal species and their metabolites mycotoxins, which are natural protective molecules for fungus itself in their habitats, allowing fungus to toxicant other organisms that may grow in the vicinity and act invasive. This study initially investigated 5 compounds that are metabolites of filamentous fungus *Aspergillus Carneus* and further focused on the compound Averufanin.

Averufanin was not only highly cytotoxic for almost all cancer cell lines in our study but also less toxic for non-tumorigenic cell lines MCF12A, HGRC1. Only cell line that had higher IC_50_ concentration than non-tumorigenic cell lines in the panel was the triple negative cell line MDA-MB-231. TNBC is the most lethal subtype of breast cancer because it has high heterogeneity and aggressive nature^28^, and lack of treatment options still remains an obstacle to overcome. Although TNBC is considered a histological subtype it also has subtypes repeatedly being described by researchers, most recent classification having five members^29^. This level of heterogeneity makes it harder to emphasize any one single differential pathway or trait and target TNBCs accordingly^30^. Same reason complicates our discussion for Averufanin’s mechanism of effecting cells. Although the lowest IC_50_ concentrations come from cell lines positive for one or more receptors among estrogen receptor (ER), progesterone receptor (PR), human epidermal growth receptor-2 (HER-2), and one is tempted to make the deduction of correlating Averufanin with receptor status, this hypothesis needs further investigation and is worth looking into in the future.

In addition to this compliant feature as an anti-cancer agent, it is also novel because it had not been studied in any field, including antioxidant, agriculture, cancer, and toxicity research. Our study shows that it is indeed a candidate for novel anti-cancer compounds however extensive studies in other fields as well can be adjuvant and elucidative. To improve the understanding of the underlying mechanisms of how Averufanin functions there can be additional experiments using different cell lines and methods in the future, allowing the compound to gain recognition and widely obtained, studied and used in the appropriate areas.

This study covers some of the pathways Averufanin affects in breast cancer cell line T47D. Obtained by the extensive western blot results, Fig. 7G demonstrates a possible mechanism of action for Averufanin on breast cancer cells represented by T47D. Averufanin induced cellular stress and DNA damage on breast cancer cells shown by γH2Ax staining. It is not uncommon for mycotoxin and their precursors to induce DNA damage, reactive oxygen species (ROS) by causing oxidative stress or alteration of mitochondrial function with unknown ways of entering or triggering cells^31^. This DNA damage resulted in the dephosphorylation of p-GSKβ in Ser9 residue^32^, allowing it to be active. Active GSK3β and possibly other DNA damage response elements^33^ increased p53 levels and phosphorylation of the upregulated tumor suppressor in Ser15 residue^34^ occurred. Both p-p53^35^ and GSK3β^15^ downregulated cell cycle regulatory protein Cyclin D1^36^, delaying passing to the S phase of cells and resulted in a cell cycle arrest at SubG1 and G1 phase, supported by the PI staining experiments. Meanwhile p-p53 and possibly other DNA damage response elements also effected DNA damage repair elements^13^ (as well as being S phase cell cycle regulatory proteins) Cyclin A2 and CDK2 and their levels elevated. Apoptosis regulator p53 also activated the caspase3/7 cascade^37,38^, as shown by the MUSE analysis, resulting in PARP cleavage and programmed cell death, apoptosis. Consequently, our data suggested that Averufanin can be contemplated and further explored as a new therapeutic strategy as an anti-cancer compound, especially for breast cancer.

## Methods

### Chemistry

#### Cultivation, isolation and structure elucidation

*Aspergillus Carneus* which was isolated from marine sponge *Agelas Oroides* previously, was cultivated in rice medium (100 g rice and 110 ml distilled water) that was autoclaved for 20 min at 121 °C before cultivation through 4 weeks. Then, medium was extracted with 500 ml ethyl acetate three times and organic solvent was dried under vacuum at 40 °C. The concentrated crude extract was fractioned between *n*-hexane and 90% aqueous methanol. Methanolic phase dried under vacuum at 40 °C and the metabolite isolation was continued with concentrated methanolic fraction^39^.

The MeOH extract from solid rice medium (1.5 g) was loaded to vacuum liquid chromatography over silica gel, eluted with a gradient of n-hexane–EtOAc (100:0 to 0:100) followed by DCM-MeOH (100:0 to 0:100) to yield 5 fractions (Fr1–Fr9). Fr1 (500 mg) was fractioned using semi-preparative RP-HPLC eluted with a gradient of MeOH-H_2_O to yield compounds Versicolorin C (5 mg), Averufanin (7 mg), Aruogosin C (3 mg), Nidurufin (3 mg) and Averufin (10 mg).

The NMR spectrums (^1^H, ^13^C) were recorded on a Bruker ARX 600. HPLC analyses were performed a Dionex ultimate 300 LC system coupled with the photodiode array detector (UVD340S). LCMSMS Semipreparative HLPC separations were carried out via he LaChrom-Merck Hitachi system (pump L7100; UV detector L7400; column Eurospher-100C18, 300 × 8 mm, Knauer, Germany) at a flow rate of 5 mL/min. LC–MS spectrums were obtained by using a Thermo Finnigan LCQ Deca mass spectrometer, and HRESIMS spectrums were carried out with a FTHRMS-Orbitrap (Thermo-Finnigan) mass spectrometer.

#### Versicolorin C

Versicolorin C, red powder; UV spectrum λ max (MeOH): 201.3, 221.1 and 289.0 nm: HRESIMS [M + H] + *m/z* 341.0655 (calcd for C_18_H_13_O_7_, 341.0655). ^1^H NMR (600 MHz, Acetone-*d*_*6*_, δ, ppm, J/Hz): 7.14 (s, H-4), 7.26 (d, H-5,* J* 2.0), 6.69 (d, H-6,* J* 2.0), 6.53 (d, H-1′,* J* 5.7), 3.62 (m, H-2′), 2.24 (m, H3′α), 1.84 (m, H-2′β), 4.14 (m, H-4′α), 4.21 (m, H-4′β). ^13^C NMR (150 MHz, Acetone-*d*_*6*_, δ, ppm); 160.1 (C-1), 126.5 (C-2), 166.0 (C-3), 109.0 (C-4), 108.0 (C-5), 164.6 (C-6), 107.5 (C-7), 164.4 (C-8), 108.9 (C-8a), 136.4 (C-5a), 189.1 (C-9), 182.4 (C-10), 107.5 (C-9a), 133.0 (C-10a), 128.1 (C-1′), 39.7 (C-2′), 27.1 (C-3′), 66.7 (C-4′)^40^.

#### Nidurifin

Nidurufin, red powder; UV spectrum λ max (MeOH): 222.6, 267.3 and 292.8 nm: HRESIMS [M + H] + *m/z* 371.0776 (calcd for C_20_H_15_O_8_, 371.0776). ^1^H NMR (600 MHz, DMSO-*d*_*6*_, δ, ppm, J/Hz): 7.13 (s, H-4), 7.26 (d, H-5, 2.4), 6.68 (d, H-7, 2.4), 5.17, (d, H-1′, 2.0), 3.70 (q, H-2′, 2.8), 1.91 (m, H-3′α), 2.31 (m, H-3′β), 1.59 (s, H-6′)^13^C NMR (150 MHz, Acetone-*d*_*6*_, δ, ppm) 159.0 (C-1), 115.2 (C-2), 160.0 (C-3), 109.4 (C-4), 108.0 (C-5), 163.2 (C-6), 105.5 (C-7), 165.0 (C-8), 189.0 (C-9), 183.5 (C-10), 133.0 (C-10a), 135.5 (C-4a), 108.9 (C-8a), 108.5 (C-1a), 80.5 (C-1′) 67.5 (C-2′), 40.2 (C-3′), 25.7 (C-4′), 101.2 (C-5′), 27.0 (C-6′)^41^.

#### Averufin

Averufin, orange powder; UV spectrum λ max (MeOH): 201.3, 221.1 and 289.0 nm: HRESIMS [M + H] + *m/z* 369.0967 (calcd for C_20_H_17_O_7_, 369.0967). ^1^H NMR (600 MHz, Acetone-*d*_*6*_, δ, ppm, J/Hz): 7.14 (s, H-4), 7.26 (d, H-5,* J* 2.0), 6.69 (d, H-6,* J* 2.0), 6.53 (d, H-1′,* J* 5.7), 3.62 (m, H-2′), 2.24 (m, H3′α), 1.84 (m, H-2′β), 4.14 (m, H-4′α), 4.21 (m, H-4′β). ^13^C NMR (150 MHz, Acetone-*d*_*6*_, δ, ppm); 160.1 (C-1), 126.5 (C-2), 166.0 (C-3), 109.0 (C-4), 108.0 (C-5), 164.6 (C-6), 107.5 (C-7), 164.4 (C-8), 108.9 (C-8a), 136.4 (C-5a), 189.1 (C-9), 182.4 (C-10), 107.5 (C-9a), 133.0 (C-10a), 128.1 (C-1′), 39.7 (C-2′), 27.1 (C-3′), 66.7 (C-4′)^42^.

#### Averufanin

Averufanin, orange powder; UV spectrum λ max (MeOH): 222.6, 267.3 and 292.8 nm: HRESIMS [M + H] + *m/z* 371.1125 (calcd for C_20_H_19_O_7_, 371.1125). ^1^H NMR (600 MHz, Acetone-*d*_*6*_, δ, ppm, J/Hz): 7.20 (s, H-4), 6.73 (d, H-5, 2.4), 7.28 (d, H-7, 2.5), 5.16 (dd, H-1′, 11.3, 2.1), 2.02 (m, H-2′α), 1.64 (m, H-2′β), 1.78 (m, H-4′α), 1.50 (m, H-4′β), 3.82 (m, H-5′), 1.32 (d, H-6′, 6.2). ^3^C NMR (150 MHz, Acetone-*d*_*6*_, δ, ppm) 111.6 (C-4), 109.0 (C-5), 109.6 (C-7), 105.8 (C-1′), 31 (C-2′), 33 (C-3′), 23.2 (C-4′), 76.5 (C-5′), 31 (C-6′)^43^.

#### Arugosin C

Arugosin C, red powder; UV spectrum λ max (MeOH): 202.7, 293.3 and 306.4 nm: HRESIMS [M + H] + *m/z* 425.1954 (calcd for C_25_H_28_O_6_, 425.1954). ^1^H NMR (600 MHz, Acetone-*d*_*6*_, δ, ppm, J/Hz): 1.72 (s, H-1), 1.72 (s, H-2), 1.70 (dd, H-4, J 6.0, 1.8), 7.40 (d, H-7 J 8.3), 6.48 (d, H-8 J 8.3), 6.87 (s, H-15), 4.20 (dd, H-19α, J 11.6, 7.9), 4.22 (dd, H-19β, J 11.6, 7.9), 2.41 (ddd, H-20, J 7.9, 5.0, 1.4), 5.33 (tdd, H-21, J 6.0, 2.9, 1.4), 1.29 (s, H-23), 1.29 (s, H-24). ^13^C NMR (150 MHz, Acetone-*d*_*6*_, δ, ppm) 18.2 (C-1), 25.0 (C-2), 132.0 (C-3), 122.5 (C-4), 23.0 (C-5), 121.0 (C-6), 133.8 (C-7), 109.1 (C-8), 157.1 (C-9), 112.0 (C-10), 162.5 (C-11), 197.2 (C-12), 151.0 (C-13), 154.2 (C-14), 116.0 (C-15), 133.0 (C-16), 148.5 (C-17), 123.8 (C-18), 60.0 (C-19), 58.5 (C-20), 72.0 (C-21), 65.0 (C-22), 28.0 (C-23), 28.0 (C-24), 17.0 (C-25)^44^.

### Biological evaluation

#### Cell culture

Cells were cultured routinely at 37 °C under 5% CO_2_ in the following mediums: OVCAR-3, OVSAHO, Kuramochi in RPMI-1640 with 10% Fetal Bovine Serum and 1% penicillin–streptomycin, MCF7, T47D, SK-BR-3, MDA-MB-231 with DMEM High Glucose with 10% Fetal Bovine Serum and 1% penicillin–streptomycin, HGRC1 with DMEM F12 Ham’s with 10% Fetal Bovine Serum and 1% penicillin–streptomycin, MCF12A with DMEM F12 Ham’s with 10% Fetal Bovine Serum, 1% penicillin–streptomycin, 10 ng/ml EGF, 500 ng/ml Hydrocortisone, 10 µg/ml Insulin.

#### NCI-60 sulforhodamine B assay for in vitro cytotoxicity screening

Cells were inoculated in 96-well plates (1000–5000 cell/well) and grown for 24 h before being treated with increasing concentrations of the compounds (0.2–100 µM or 0.1–50 µM). After 72 h and 48 h of incubation, cells were fixed with 10% (v/v) trichloroacetic acid for 1 h at 4 °C. Then plates were washed five times with deionized water and left to dry. Next day, cells were stained with 0.4% (m/v) of Sulforhodamine B in 1% acetic acid solution in the dark at room temperature for 30 min. The plates washed five times with 1% acetic acid before air-drying. Bound SRB was dissolved in 10 mM Tris-base solution. The absorbance was read in a plate reader at 515 nm^45^. DMSO was used as a negative control. The experiment was performed in triplicates.

#### Cell cycle analysis with PI staining and flow cytometry

In order to investigate the effect of the compound on cell cycle, cells were plated (1 × 10^5^ cells) into flat bottom 6-well plates and treated with both IC_50_ and IC_75_ concentrations. After 48 h treatment cells were collected, washed with ice-cold DPBS and fixed with ethanol (70%) with constant gentle vortexing. Then cells were centrifuged and resuspended in PI solution (50 µg/ml propidium iodide (Sigma Aldrich), 0.1 mg/ml RNAseA and 0.05% TritonX-100 in ice-cold DPBS) and incubated at 37 °C for 40 min. Samples were washed with DPBS and re-suspended. Flow Cytometry (CytoFLEX) was used for analysis.

#### MUSE annexin V assay

6-well plates were used for cell inoculation (1 × 10^5^ cell/well), and then cells were grown for 24 h and treated with IC_50_ concentrations of the compound and DMSO control. After 72 h treatment, cells were collected and diluted with 1% FBS/PBS solution to 100/500 cell/µl. 75 µl cell + 75 µl Annexin V dye (Luminex, MUSE) were incubated at room temperature in the dark for 20 min before analyzing with MUSE Cell Analyzer^46^.

#### MUSE Caspase3/7 assay

Human breast cancer cells were plated in 6-well plates (1 × 10^5^ cell/well) and grown for 24 h and treated with IC_75_ concentrations of the corresponding compound and DMSO controls. After 48 h treatment, cells were collected and diluted with 1% FBS/PBS solution to 300–500 cell/µl. Results were analyzed using MUSE Caspase 3/7 kit according to the manufacturer’s protocol^47^.

#### Immunofluorescence staining assay for γH2Ax

In order to investigate the effect of Averufanin on DNA damage, cells were inoculated in 6-well plates with a coverslip in the bottom. (1.5 × 10^5^ cells/well) 24 h later they were treated with IC_50_, IC_75_ concentrations of the compound or DMSO control for 48 h. The medium was removed and coverslip was washed with DPBS three times. 4% Paraformaldehyde in 1× DPBS solution was given to each well to enough to cover the surface. It was incubated for 20 min in RT in the dark before DBPS wash for three times again. Later 0.1% Triton-X 100 DPBS solution was given to cells for 15 min in RT in the dark. The permeabilization step was followed by a wash step with 0.1% Tween 20 DPBS solution. Blocking was performed with 5% Blotting Grade Blocker in DPBS for 30 min in RT. Coverslips were incubated with the primary antibody γH2Ax (Cell Signaling, Denvers, MA, USA) overnight at 4 °C, in 1:100 dilution with DPBS. Next day, secondary antibody incubation with Alexa Flour 594 was applied for 2 h in RT. (1:100 dilution). Mounting medium containing DAPI (Vectashield) was applied to slides and coverslips were left to dry overnight at RT in the dark. Next day, coverslips were fixed on the slides with varnish and Confocal Microscopy (Leica, with LAS X Software) was used to visualize the samples. Subvolume sizes of x, y dimensions for control (580.68 µm × 1160.8 µm), IC_50_ (870.74 µm × 580.68 µm) and IC_75_ (870.74 µm × 870.74 µm) were gathered and different crop regions were cut to analyze with ImageJ and GraphPad PRISM.

#### Western blotting

Cells were cultured in 15-cm culture dishes (5 × 10^6^ cells/dish) for 24 h and treated with IC_50_, IC_75_ concentrations of the compounds or DMSO control for 48 h. Pellets were collected and lysed with 1× RIPA Lysis Buffer, 10% Proteinase Inhibitor and 1% Phosphatase Inhibitor. Lysates were incubated on ice for 45 min, vortexing every 15 min. Samples were centrifuged in 13000×G for 15 min. The protein concentration of the lysates was determined with the BCA assay (Thermo Scientific, 23225 Kit Protocol was followed). Cell lysates containing 20 µg protein were mixed with 4X Laemmli Sample Buffer, 1/6 β-Mercaptoethanol and denatured for 6 min in 95 °C. Samples were loaded to BIORAD Precast gels of 15 wells (4–15%). After electrophoresis, the proteins were transferred to PVDF membranes (BIORAD) with Semidry Transfer System (BIORAD), followed by overnight incubation in blocking solution (5% Blotting Grade Blocker in 1× TBS-T (0.2% tween)). PARP (Cell signaling), p53 (Cell Signaling), Phospho-p53 (Ser15) (Cell Signaling), GSK3β (Cell Signaling), phosphor-GSK3β (Ser9) (Cell Signaling), CyclinD1 (Cell Signaling), Cyclin A2 (Cell Signaling), CDK2 (Cell Signaling) primary antibodies were used in a ratio of 1:500 to 1:1000 in 5% BSA-TBS-T, incubated for 1 h in room temperature. Secondary antibodies, anti-rabbit (Sigma Aldrich), anti-mouse (abcam), were used in 1:2500 dilutions in 5%Blotting Grade Blocker-TBS-T for 1 h at room temperature. β-Actin (abcam), Vinculin (Cell Signaling) or GAPDH (abcam) primary antibody for equal loading analysis was used in 1:1000 dilution in 5% Blotting Grade Blocker in TBS-T for 1 h at room temperature. For visualization of the results, chemiluminescence was performed with ECL. The membranes were visualized using LI-COR Fc. Sample bands were visualized with Chemi light exposed for 10 min and 700 light exposed for 1 min for the protein standards (ladder). All proteins were run in the same gel with their loading controls and only physical application was vertical cropping. All blots were evenly treated with brightness and contrast modifications (mostly + %20 Contrast and + %20 brightness of Office Powerpoint was applied.) and no bands were exaggerated or lost during the processing. To quantitatively analyze the band intensities, ImageJ was uses, statistical analysis were done with GraphPad PRISM.

### Statistical analysis

Statistical analysis was performed with GraphPad PRISM encoded tests. Most of the data was analyzed with unpaired two-tailed t-test and also significancy of compound treated groups were validated with one-way ANNOVA test comparing to DMSO treatment groups. For (*) p < 0.05, (**) p < 0.01, (***) p < 0.001 and (****) p < 0.0001.

## Supplementary Information

Supplementary Information.

## Data Availability

All data generated or analyzed during this study are included in this published article.
