# Supplementary material for: Aspergillus Carneus metabolite Averufanin induced cell cycle arrest and apoptotic cell death on cancer cell lines via inducing DNA damage
Source: Sci Rep. 2023 Apr 20;13:6460. doi: 10.1038/s41598-023-30775-w (PMC10119153; doi:10.1038/s41598-023-30775-w)
Supplement: Supplementary file 1 — Supplementary Information. [file 41598_2023_30775_MOESM1_ESM.pptx]

## Slide 1
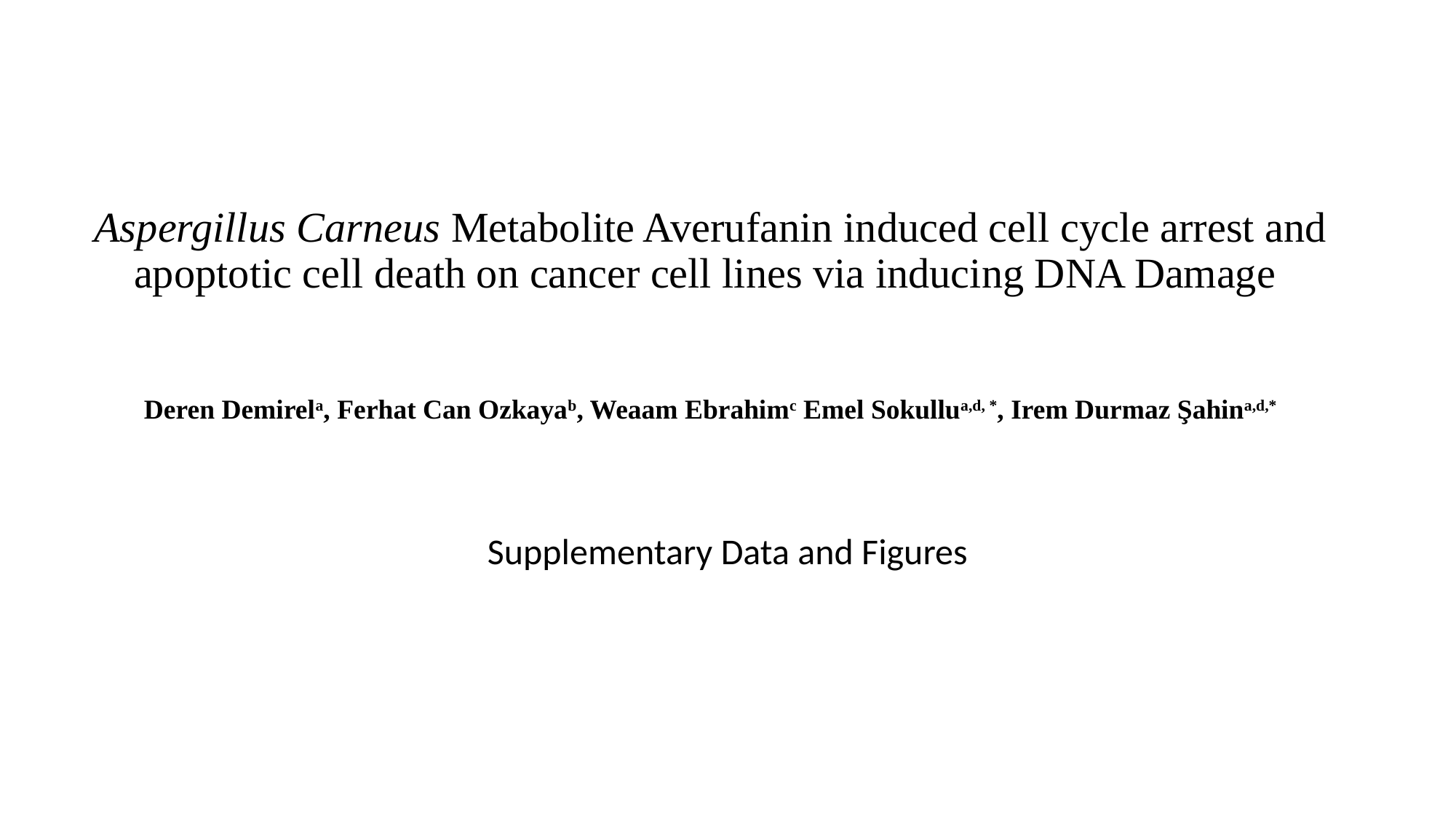

# Aspergillus Carneus Metabolite Averufanin induced cell cycle arrest and apoptotic cell death on cancer cell lines via inducing DNA Damage  Deren Demirela, Ferhat Can Ozkayab, Weaam Ebrahimc Emel Sokullua,d, *, Irem Durmaz Şahina,d,*
Supplementary Data and Figures

## Slide 2
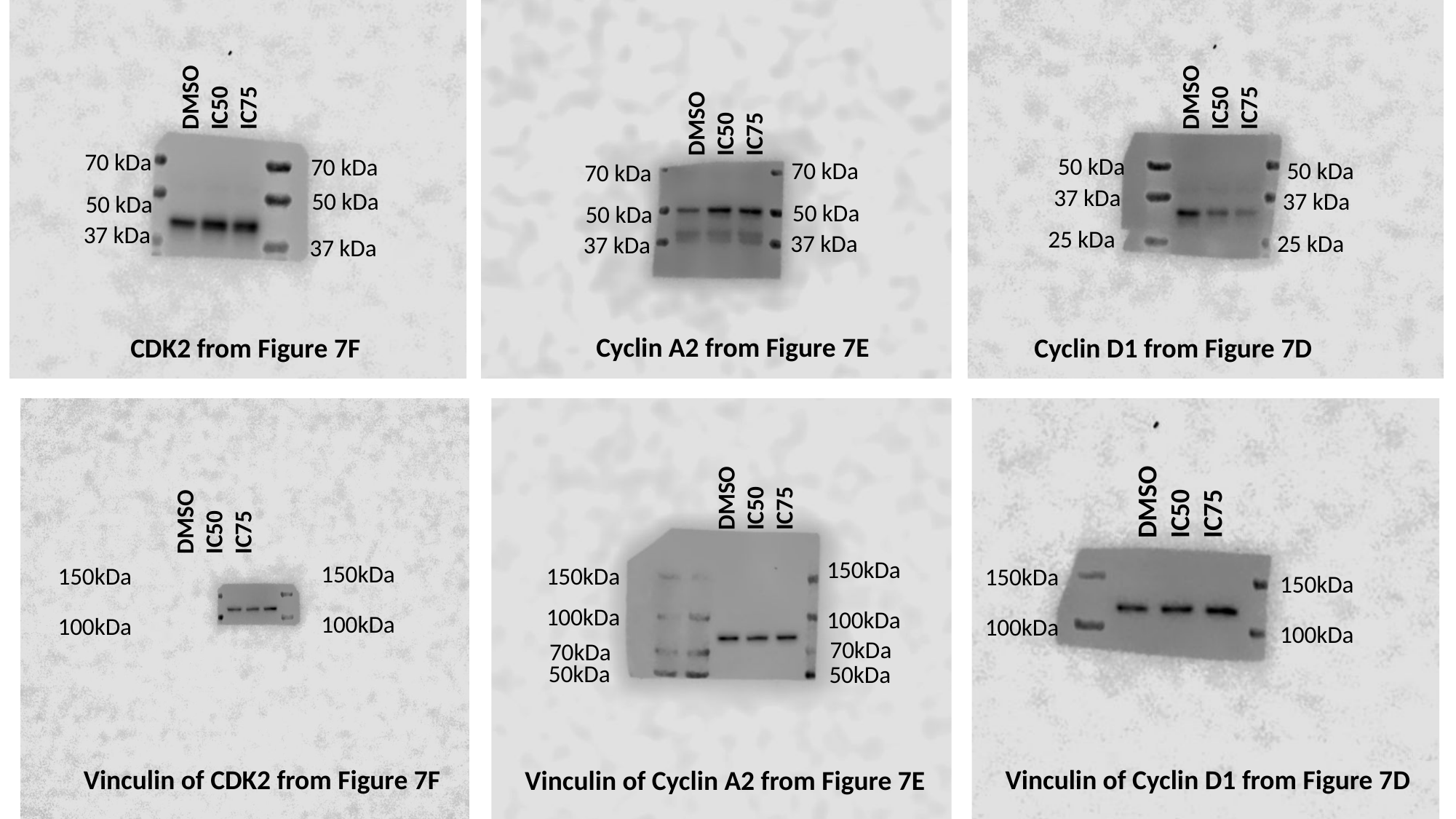

DMSO
IC50
IC75
DMSO
IC50
IC75
DMSO
IC50
IC75
70 kDa
50 kDa
70 kDa
50 kDa
70 kDa
70 kDa
37 kDa
37 kDa
50 kDa
50 kDa
50 kDa
50 kDa
37 kDa
25 kDa
37 kDa
25 kDa
37 kDa
37 kDa
Cyclin A2 from Figure 7E
CDK2 from Figure 7F
Cyclin D1 from Figure 7D
DMSO
IC50
IC75
DMSO
IC50
IC75
DMSO
IC50
IC75
150kDa
150kDa
150kDa
150kDa
150kDa
150kDa
100kDa
100kDa
100kDa
100kDa
100kDa
100kDa
70kDa
70kDa
50kDa
50kDa
Vinculin of CDK2 from Figure 7F
Vinculin of Cyclin D1 from Figure 7D
Vinculin of Cyclin A2 from Figure 7E

## Slide 3
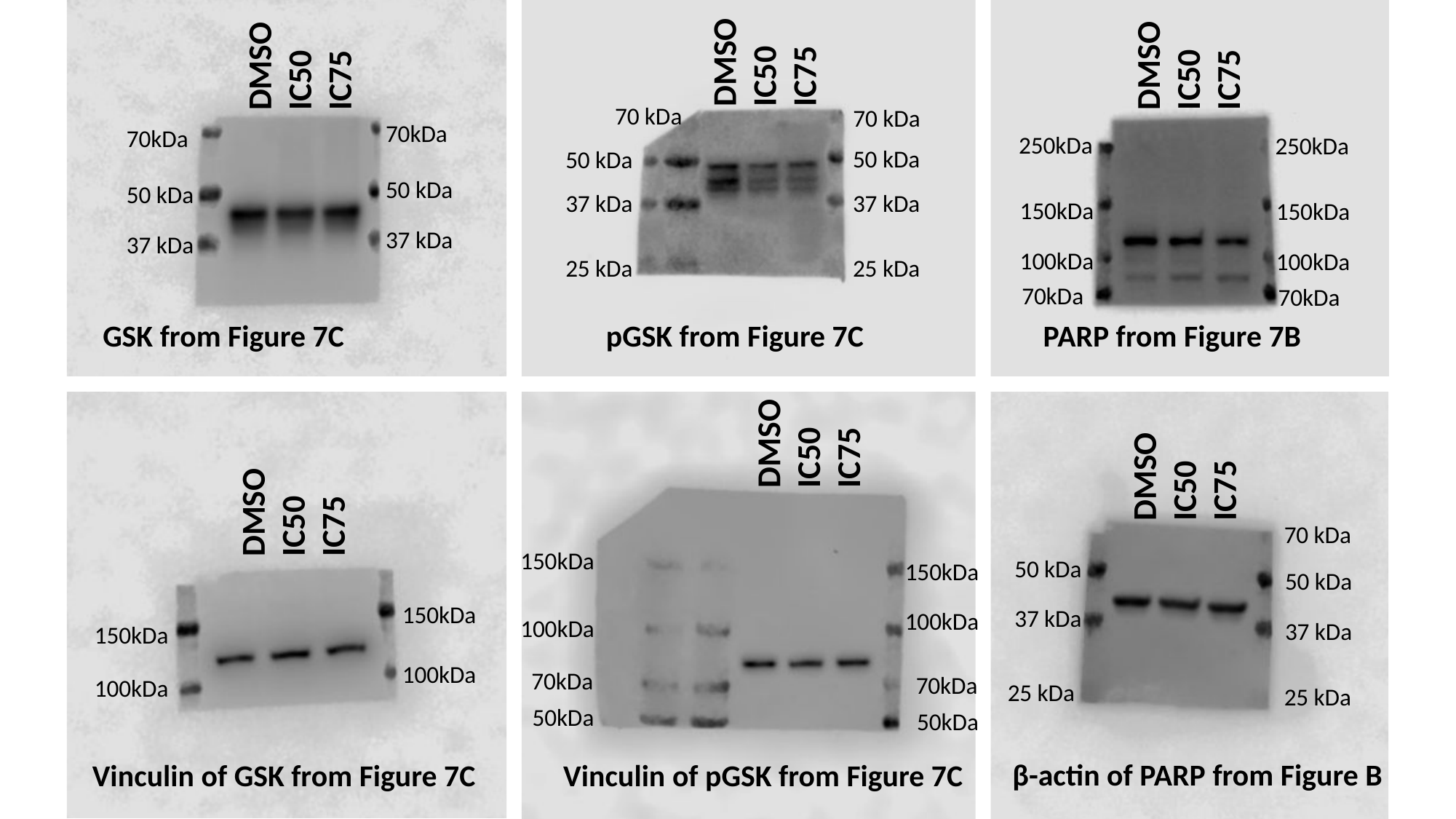

DMSO
IC50
IC75
DMSO
IC50
IC75
DMSO
IC50
IC75
70 kDa
70 kDa
70kDa
70kDa
250kDa
250kDa
50 kDa
50 kDa
50 kDa
50 kDa
37 kDa
37 kDa
150kDa
150kDa
37 kDa
37 kDa
100kDa
100kDa
25 kDa
25 kDa
70kDa
70kDa
GSK from Figure 7C
pGSK from Figure 7C
PARP from Figure 7B
DMSO
IC50
IC75
DMSO
IC50
IC75
DMSO
IC50
IC75
70 kDa
150kDa
50 kDa
150kDa
50 kDa
150kDa
37 kDa
100kDa
100kDa
37 kDa
150kDa
100kDa
70kDa
70kDa
100kDa
25 kDa
25 kDa
50kDa
50kDa
β-actin of PARP from Figure B
Vinculin of GSK from Figure 7C
Vinculin of pGSK from Figure 7C

## Slide 4
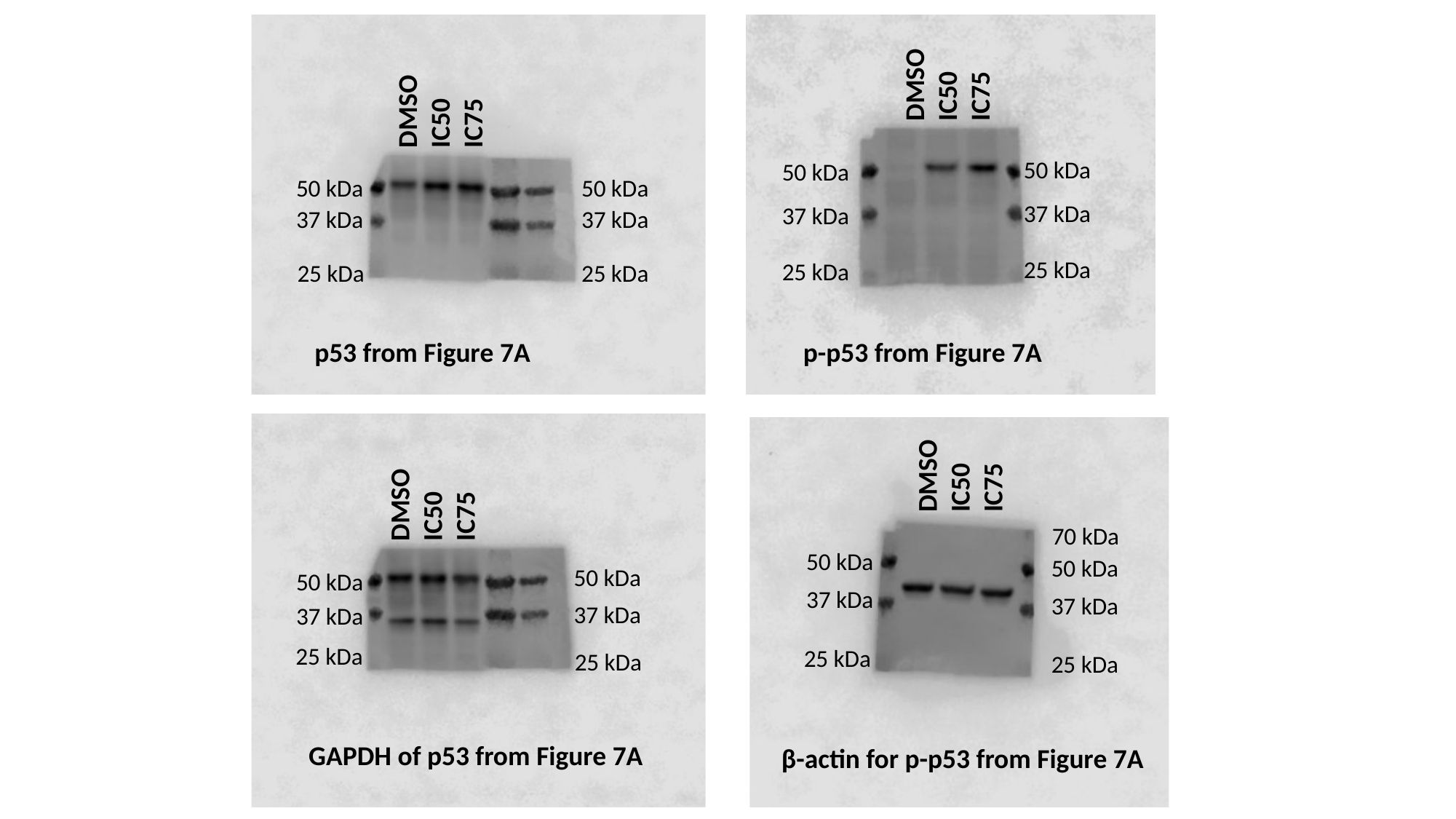

DMSO
IC50
IC75
DMSO
IC50
IC75
50 kDa
50 kDa
50 kDa
50 kDa
37 kDa
37 kDa
37 kDa
37 kDa
25 kDa
25 kDa
25 kDa
25 kDa
p53 from Figure 7A
p-p53 from Figure 7A
DMSO
IC50
IC75
DMSO
IC50
IC75
70 kDa
50 kDa
50 kDa
50 kDa
50 kDa
37 kDa
37 kDa
37 kDa
37 kDa
25 kDa
25 kDa
25 kDa
25 kDa
GAPDH of p53 from Figure 7A
β-actin for p-p53 from Figure 7A
